# FAP-targeted CAR-T suppresses MDSCs recruitment to improve the antitumor efficacy of claudin18.2-targeted CAR-T against pancreatic cancer

**DOI:** 10.1186/s12967-023-04080-z

**Published:** 2023-04-12

**Authors:** Yifan Liu, Yansha Sun, Peng Wang, Songling Li, Yiwei Dong, Min Zhou, Bizhi Shi, Hua Jiang, Ruixin Sun, Zonghai Li

**Affiliations:** 1 State Key Laboratory of Oncogenes and Related Genes, Shanghai Cancer Institute, Renji Hospital, Shanghai Jiaotong University School of Medicine, No. 25/Ln2200 XieTu Road, Shanghai, 200032 China; 2 State Key Laboratory for Oncogenes and Related Genes, Renji-Med X Clinical Stem Cell Research Center, Ren Ji Hospital, School of Biomedical Engineering, Shanghai Jiao Tong University, Shanghai, 200030 China; 3 CARsgen Therapeutics, Shanghai, 200032 China

## Abstract

**Purpose:**

The claudin 18.2 (CLDN18.2) antigen is frequently expressed in malignant tumors, including pancreatic ductal adenocarcinoma (PDAC). Although CLDN18.2-targeted CAR-T cells demonstrated some therapeutic efficacy in PDAC patients, further improvement is needed. One of the major obstacles might be the abundant cancer-associated fibroblasts (CAFs) in the PDAC tumor microenvironment (TME). Targeting fibroblast activation protein (FAP), a vital characteristic of CAFs provides a potential way to overcome this obstacle. In this study, we explored the combined antitumor activity of FAP-targeted and CLDN18.2-targeted CAR-T cells against PDAC.

**Methods:**

Novel FAP-targeted CAR-T cells were developed. Sequential treatment of FAP-targeted and CLDN18.2-targeted CAR-T cells as well as the corresponding mechanism were explored in immunocompetent mouse models of PDAC.

**Results:**

The results indicated that the priorly FAP-targeted CAR-T cells infusion could significantly eliminate CAFs and enhance the anti-PDAC efficacy of subsequently CLDN18.2-targeted CAR-T cells in vivo. Interestingly, we observed that FAP-targeted CAR-T cells could suppress the recruitment of myeloid-derived suppressor cells (MDSCs) and promote the survival of CD8^+^ T cells and CAR-T cells in tumor tissue.

**Conclusion:**

In summary, our finding demonstrated that FAP-targeted CAR-T cells could increase the antitumor activities of sequential CAR-T therapy via remodeling TME, at least partially through inhibiting MDSCs recruitment. Sequential infusion of FAP-targeted and CLDN18.2-targeted CAR-T cells might be a feasible approach to enhance the clinical outcome of PDAC.

**Supplementary Information:**

The online version contains supplementary material available at 10.1186/s12967-023-04080-z.

## Introduction

Pancreatic ductal adenocarcinoma (PDAC), an aggressive and lethal cancer with 5-year overall survival of 10%, is the seventh leading cause of cancer death worldwide [1, 2]. Most patients are diagnosed in the advanced stage too late for curable operation; thus, systemic therapy is essential [1]. As living drugs, chimeric antigen receptor (CAR-T) therapy has recently demonstrated remarkable results for hematological malignancies [3]. This emerging immunotherapy has also drawn significant attention to solid cancers. Previously we had developed claudin18.2 (CLDN18.2)-targeted CAR-T cells, which recognize one tight junction protein highly expressed in stomach and pancreatic cancer [4]. Preliminary data from phase I clinical trial (NCT03874897) demonstrated that CLDN18.2-targeted CAR-T cells were safe and showed some antitumor activities in advanced gastrointestinal cancer, including pancreatic cancer [5]. To further improve the efficacy of CLDN18.2-targeted CAR-T therapies in PDAC, new therapeutic strategies are urgently needed. One of the main impediments encountered in CAR-T therapies for solid tumors is the tumor microenvironment (TME), especially in pancreatic cancer [6]. Although CLDN18.2-targeted CAR-T cells could effectively attack CLDN18.2-positive tumor cells in vitro, its accumulation is limited in the pancreatic cancer mouse model [7]. Increasing evidence reveals that PDAC TME is featured by poor immune infiltration and active stroma, including fibroblasts, tumor vasculature, immune cells, and extracellular matrix (ECM) [8]. Significantly, many studies highlight the contribution of PDAC stroma in immune escape and poor prognosis [8, 9]. Specifically, several physical and biological barriers prevent CAR-T from trafficking and infiltrating the tumor site. Moreover, the immunosuppressive microenvironment impedes the function and persistence of CAR-T cells [6, 10]. Thus, to boost the antitumor response of CLDN18.2-targeted CAR-T therapy in PDAC, one strategy is to reshape the TME.

Cancer-associated fibroblasts (CAFs), a fundamental component of PDAC stroma, maintain several critical functions in TME[11]. Particularly, CAFs deposit and remodel ECM can form a barrier to drug delivery and immune surveillance. In addition, CAFs establish immune crosstalk by secreting chemokines and cytokines such as IL-6, TGF-β, and CXCL12, thereby interfering with T cell function and recruiting myeloid-derived suppressor cells (MDSC), regulatory T cells (Treg) and tumor-associated macrophage (TAM) [11, 12]. Therapeutically, a previous study found that depletion of CAFs can recover the antitumor effects of CTLA-4 and PD-L1 in an autochthonous PDAC model [13]. Therefore, considering that CAFs serve as a physical barrier and immunosuppressor, targeting CAFs may augment antitumor immune response, especially in the fibroblast-rich tumor [14].

Expression of fibroblast activation protein (FAP) is a vital characteristic of active CAFs, including pancreatic cancers [15]. The safety and efficiency of systemic FAP-targeted therapy have been demonstrated by whole-body organ immunohistochemistry (IHC) and other immunotherapies [16–19]. However, the results of this target CAR-T studies were contradictory. One previous study revealed that FAP-targeted CAR-T cells show trivial antitumor effects and induces severe toxicity [20]. In contrast, several other preclinical studies, including mesothelioma, breast cancer, and colon and lung adenocarcinoma, have shown FAP-targeted CAR-T cells control tumor growth or prolonged survival without obvious toxicity [21–23]. It has been reported that FAP-targeted CAR-T cells could induce endogenous T cells infiltration and activation in tumor tissue [22]. Nevertheless, the antitumor effect of FAP-targeted CAR-T cells alone is limited or temporary [21, 22]. FAP-targeted CAR-T cells demonstrated enhanced antitumor response when combined with other immune therapies, including tumor-associated-antigen (TAA) CAR-T or vaccine [21, 22]. FAP-targeted CAR-T cells may be used as a TME regulator for additional immune therapy, such as CLDN18.2-targeted CAR-T cells.

In this study, we developed one kind of novel FAP-targeted CAR-T cells. It is worth noting that CD8^+^ T cell augmentation occurs about one week after FAP-targeted CAR-T cells infusion rather than the first three days [21, 22]. Thus, a sequential treatment plan in which infused FAP-targeted CAR-T cells priorly and CLDN18.2-targeted CAR-T cells one week late was settled to improve the antitumor effect of two different CAR-T cells. We investigated whether CAFs could be eliminated in tumor sites by FAP-targeted CAR-T cells and further improve the antitumor ability of CLDN18.2-targeted CAR-T cells by TME remodeling, aiming to develop an effective and safe pancreatic cancer treatment strategy.

## Materials and methods

### Data sources, processing, and analysis methods of bioinformatics part

The Cancer Genome Atlas (TCGA) gene expression data of solid cancer tissues were obtained from the cBioPortal for Cancer Genomics (https://www.cbioportal.org/) [24]. Fragments per kilobase of exon per million reads mapped (FPKM) were used for expression quantification, then normalized by log transform. The ESTIMATE algorithm was applied to estimate the tumor purity score [25]. By R package “maxstat”, the optimal cutoff of FAP expression was identified for significantly grouping TCGA-PDAC patients [26]. Log-rank tests compared the survival outcomes of the two groups (high-FAP *vs.* low-FAP). Linear models were used to identify differentially expressed genes (DEGs) between the two groups using an R package “limma“ [27]. A false discovery rate (FDR) adjusted *p-value* < 0.001 and absolute value of log2 (fold change) > 1.5 was set as the threshold for DEGs. Gene Set Enrichment Analysis (GSEA) of hallmark gene sets from MSigDB database v7.4 was performed by GSEA software 4.1.0 and FDR *q-value* < 0.25 were considered significantly enriched. [28]. Single Sample Gene Set Enrichment Analysis (ssGSEA) of Immune Suppressive and Fibroblast signatures obtained from IOBR (Immune Oncology Biological Research) was calculated by R package “IOBR” [29]. The protein expression of FAP in tumor tissues was obtained from HPA database (Human Protein Atlas, https://www.proteinatlas.org/) [30]. All bioinformatic analyses were performed with R version 4.0.2 and its appropriate packages.

### Cell line and medium

Murine pancreatic carcinoma cell lines PANC02 (obtained from ATCC) and KPC1199 (KPC mice derived PDAC cell line FC1199, a gift from Professor Jing Xue) were cultured in DMEM (Gibco) with fetal bovine 10% FBS (Gibco). PANC02 were transduced using *p*WPT-CLDN18.2 lentiviral vectors to stable express murine CLDN18.2 (designated as PAN02-A2). Murine breast cancer cell line E0771 cells (EGFR*v*III, gifted by Dr. Xiang Zhang of Baylor College of Medicine) were cultured in RPMI 1640 medium (Gibco) with 10% fetal bovine serum (FBS) (Gibco). Murine fibroblast cell line NIH 3T3 cell lines (obtained from ATCC) were cultured in DMEM (Gibco) with 10% newborn calf serum (NCS). NIH 3T3 was transduced to stably express murine FAP using *p*WPT-GFP- mFAP lentiviral vectors (designated as 3T3-mFAP). Human fibrosarcoma cell line HT-1080 (obtained from ATCC) was cultured in DMEM (Gibco) with 10% fetal bovine serum (FBS) (Gibco). 3T3-mFAP (Flag) and HT1080-huFAP were also established by transient transfection using *p*WPT-mFAP-Flag and *p*WPT-huFAPα-Flag lentiviral vectors, respectively.293T packaging cell lines (obtained from the ATCC) were cultured in DMEM (Gibco, USA) with 10% FBS (Gibco). All cells were routinely maintained at 37 °C in a 5% CO_2_ atmosphere incubator.

### CAR design and generation of CAR-T

Anti-FAP antibody was selected from a naïve scFv phage library. Then the antibody was converted into scFv-Fc format, transiently expressed by 293F cells and purified using protein A affinity chromatography. The binding specificity to 3T3-mFAP and HT1080-huFAP was investigated by flow cytometry. The binding affinity to FAP was tested by Biacore T200 (GE Healthcare). The 8E3-mBBZ CAR construct was composed of 8E3 scFv (FAP Antibody) linked by the hinge and transmembrane region of the CD8α chain and intracellular 4-1BB and CD3ζ. The 8E5 scFv (CLDN18.2 Antibody) linked to the hinge and transmembrane regions of the murine CD8α chain and intracellular murine 4-1BB, and CD3ζ signaling domains generated the 8E5-mBBZ CAR. 806-28z CAR was constructed by 806 scFv (EGFR*v*III antibody) linked to mouse CD28 and CD3-ζ endo-domain. 293T cells were transfected with the CAR-expressing plasmid and the retroviral packaging plasmid *p*CL-Eco (Addgene) using polyethyleneimine. Retroviral supernatant was collected 48 h later and filtered through a 0.45 μm syringe (Millipore). T cells from C57BL/6 mice spleen were sorted by isolation kit (STEMCELL Technologies) and stimulated with anti-mouse CD3/CD28 magnetic beads (Thermo Fisher Scientific) for 24 h, and then infected with retrovirus in RetroNectin (Takara)-coated plates.

### In vivo **animal experiment**

All animal procedures were approved by the Experiment Animal Care Commission of the Shanghai Cancer Institute and performed according to the protocol. The laboratory animal ethics review ID was 179166. Female C57BL/6 mice were bought from Shanghai Sippr BK laboratory and housed under specific pathogen-free conditions. In the experimental fibroblast-rich model, EGFR*v*III-positive E0771 tumor cells (2 × 10^6^) alone or combined with NIH 3T3 fibroblast (2 × 10^6^ or 4 × 10^6^) were in situ inoculated into the fourth inguinal mammary fat pads. The CPA (100 mg/kg) was administered *i.p.* 24 h before 2 × 10^6^ 806-28Z or UTD (untransduced) T cells injection (day 14). In the PANC02 or KPC1199 pancreatic cancer models, C57BL/6 mice were inoculated subcutaneously with CLDN18.2-positive PANC02 or KPC1199 tumor cells (2 × 10^6^) on the right flank on day 0, respectively. CPA (100 mg/kg) was administered *i.p.* 24 h before 2 × 10^6^ CAR-T or UTD T cells’ injection (First Injection). 7 days later, 2 × 10^6^ CAR-T or UTD T were sequentially injected (Second Injection). Tumor dimensions and body weight were measured every 2–5 days. Tumor volume was measured by caliper and calculated by the formula V = (length * width^2^)/2. In addition, tumor inhibition ratio was calculated by the formula Tumor Inhibition Ratio= (UTD.Volume-Treatment.volume)/ UTD.Volume.

### Flow cytometry

To evaluate CAR expression, cells were incubated for an hour with biotin-conjugated goat-anti-human Fab (Jackson ImmunoResearch) and then incubated with a PE-conjugated anti-streptavidin antibody for 30 min. To measure the expression of CLDN18.2 on tumor cells, cells were incubated with h8E5(2I) antibody for an hour, which our lab generated, followed by incubating with fluorescein isothiocyanate (FITC)-conjugated goat anti-human antibody (Invitrogen) for 30 min. The analysis was performed by using a flow cytometer. For in vivo detection of tumor-infiltrating immune cells, tumor tissue isolated from tumor-bearing mice was cut into small pieces and resuspended in medium containing collagenase type IV (0.5 mg/mL, Sigma Aldrich,), collagenase type I (0.5 mg/mL, Sigma Aldrich), hyaluronidase (0.5 mg/mL, Sigma Aldrich), and DNase I (0.02 mg/mL, StemCell Technology) for 30 min at 37 °C for digestion. The suspensions were filtered through a 70 μm Falcon cell strainer, centrifuged, and stained with antibodies against CD3ε, CD4, CD8, PD1, LAG3, TIM3, PD-L1, CD49b, CD45, CD11b, MHCII, F4-80 and Ly6G according to the manufacturer’s instructions. Detailed flow cytometry gating strategy can be found in Additional file 1: Fig.S6g.

### In vitro cytotoxicity assays

To study the cytotoxicity of CAR-T cells, T cells were cocultured with tumor cells at effector-to-target ratios of 3:1, 1:1, and 1:3 in 96-well plates. After 18 h, the specific cytotoxicity of T cells was analyzed by the lactate dehydrogenase release in the supernatants using the Cytotoxicity Detection Kit (Roche).

### Immunohistochemistry (IHC) staining

Tumor tissues and organs were fixed with formalin, embedded in paraffin, and sectioned at a 5-mm thickness. The organs were stained with hematoxylin and eosin. The tumor tissue sections were examined by IHC staining by anti-α-SMA (CST), anti-CD4 (Abcam) and anti-CD8α (Abcam). Briefly, the sections were exposed to 3% H_2_O_2_ in methanol after deparaffinization and rehydration and then blocked with 1% BSA for 30 min at room temperature. After blocking, the sections were incubated with primary antibody overnight at 4 °C, followed by peroxidase-conjugated secondary antibodies (ChemMate DAKO EnVision Detection Kit) and detection reagents. CD4^+^ and CD8^+^ cells were quantified by measuring the number of stained cells in sections from three mice in each group.

### Statistical analysis

All data are presented as the mean ± SEM. Student’s t test was used for two-sample comparisons. *One-way analysis of variance* (ANOVA) was used for multi-sample comparisons. Tumor growth data were analyzed with *two-way ANOVA*. All statistical analyses were done using GraphPad Prism 9.0 software. *p < 0.05, **p < 0.01, and ***p < 0.001 were considered statistically significant.

## Results

### FAP is highly expressed in pancreatic cancer and is strongly associated with immune suppression

**Fig. 1:**
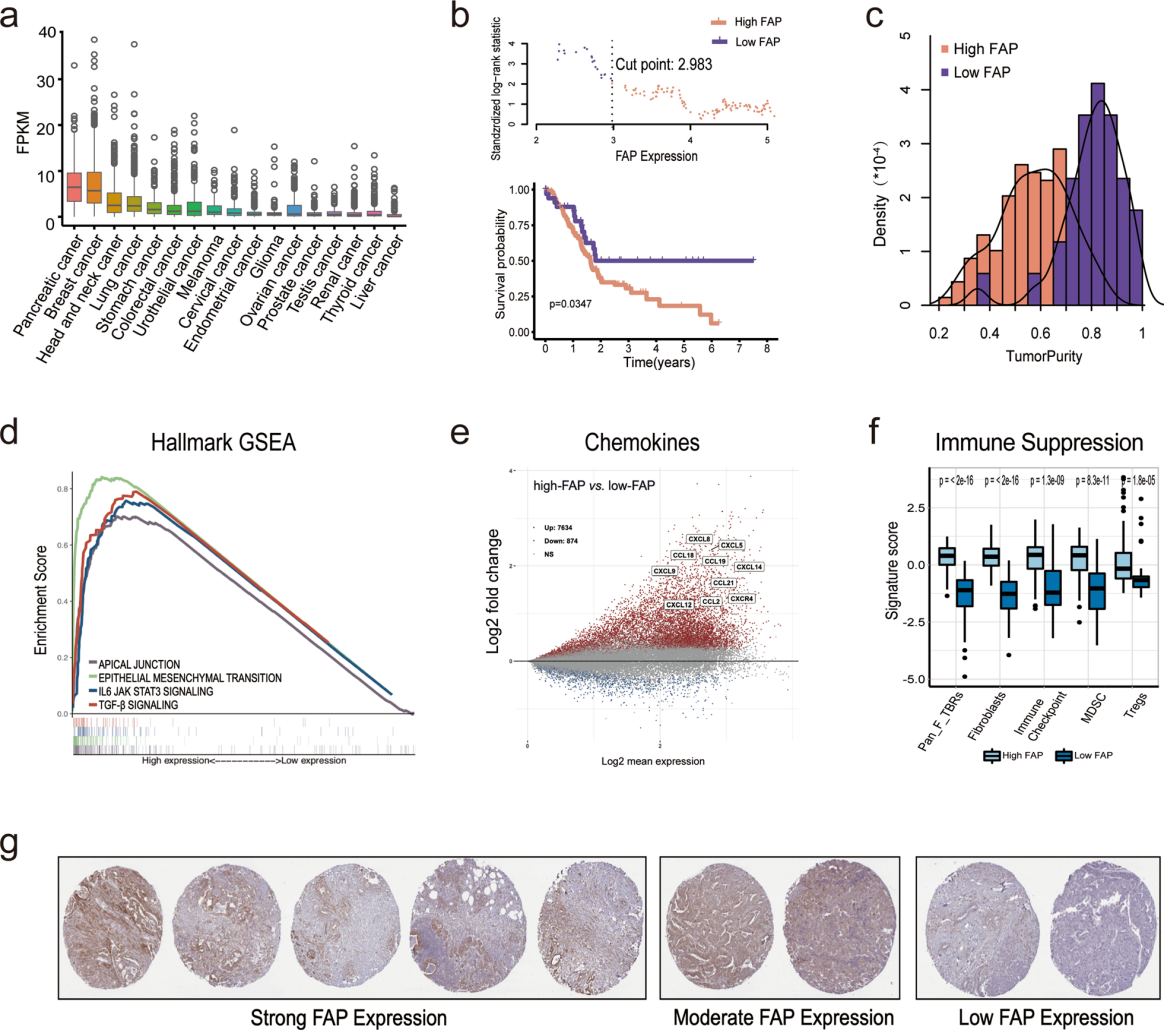
Bioinformatics analysis of FAP biological and tumor microenvironment characteristics in pancreatic cancer. ** a** Pan-cancer analysis of FAP expression. **b** Identification of optimal FAP expression cutoff dividing PDAC cohort. The upper scatter plot shows each cutoff point’s standardized log-rank statistic value. The lower Kaplan-Meier plot shows overall survival for patients divided by the optimal cutoff. **c**–**f** Biology and TME characteristics in high-FAP* vs.* low-FAP groups of PDAC cohort. **c** Density distribution of tumor purity. **d** Enrichment score of HALLMARK gene-sets related to high FAP expression. **e** Volcano map of the chemokines. **f** Immune suppressive characteristics estimated by IOBR. **g** IHC staining of FAP protein expression in 9 pancreatic cancer samples

First, we investigate the expression of FAP among 19 common solid tumors from the TCGA database. FAP expression was significantly up-regulated in pancreatic and breast cancer, indicating that FAP-positive cells were abundant in these two cancers (Fig. 1a). To identify the optimal FAP cutoff level for grouping PDAC patients significantly, the survival outcomes of all possible cutoff level was compared by log-rank tests. And then, the expression level of 2.9, nearest to the FAP expression median and with a significant survival difference, was identified as the optimal cutoff level (Fig. 1b). Thus, PDAC patients were divided into high-FAP and low-FAP groups. By the ESTIMATE algorithm, patients in high-FAP yielded a lower tumor purity than those in low-FAP groups, revealing a complicated and abundant stromal was involved in those patients (Fig. 1c). Besides, we also observed the activation of immune checkpoint and other immunosuppressive cells, including MDSCs, Treg, and fibroblasts in the high-FAP group (Fig. 1f, Additional file 1: Fig.S1a-b). Given the poor survival of high-FAP patients, these data revealed that effective antitumor immune response was suppressed in those patients. Furthermore, the results of DEGs and HALLMARK GSEA analysis showed that the FAP high expression was significantly correlated with EMT, cell junction, IL-6/STAT3, and TGF-β signaling pathway and chemokine releasing, which further suggests its immune suppressive role and close interaction with tumor (Fig. 1d–e). Then, IHC analysis of FAP in the pancreatic from the HAP database was analyzed. In 7 of 9 patients, we found a large amount of CAFs around tumor cells, with moderate or high FAP staining (Fig. 1g). Several tumors also showed a similar phenomenon, suggesting that FAP is a potential target for fibroblast-rich tumors, including pancreatic cancer (Additional file 1: Fig.S1c).

### CAFs impair the antitumor activity of CAR-T cells in vivo

**Fig. 2:**
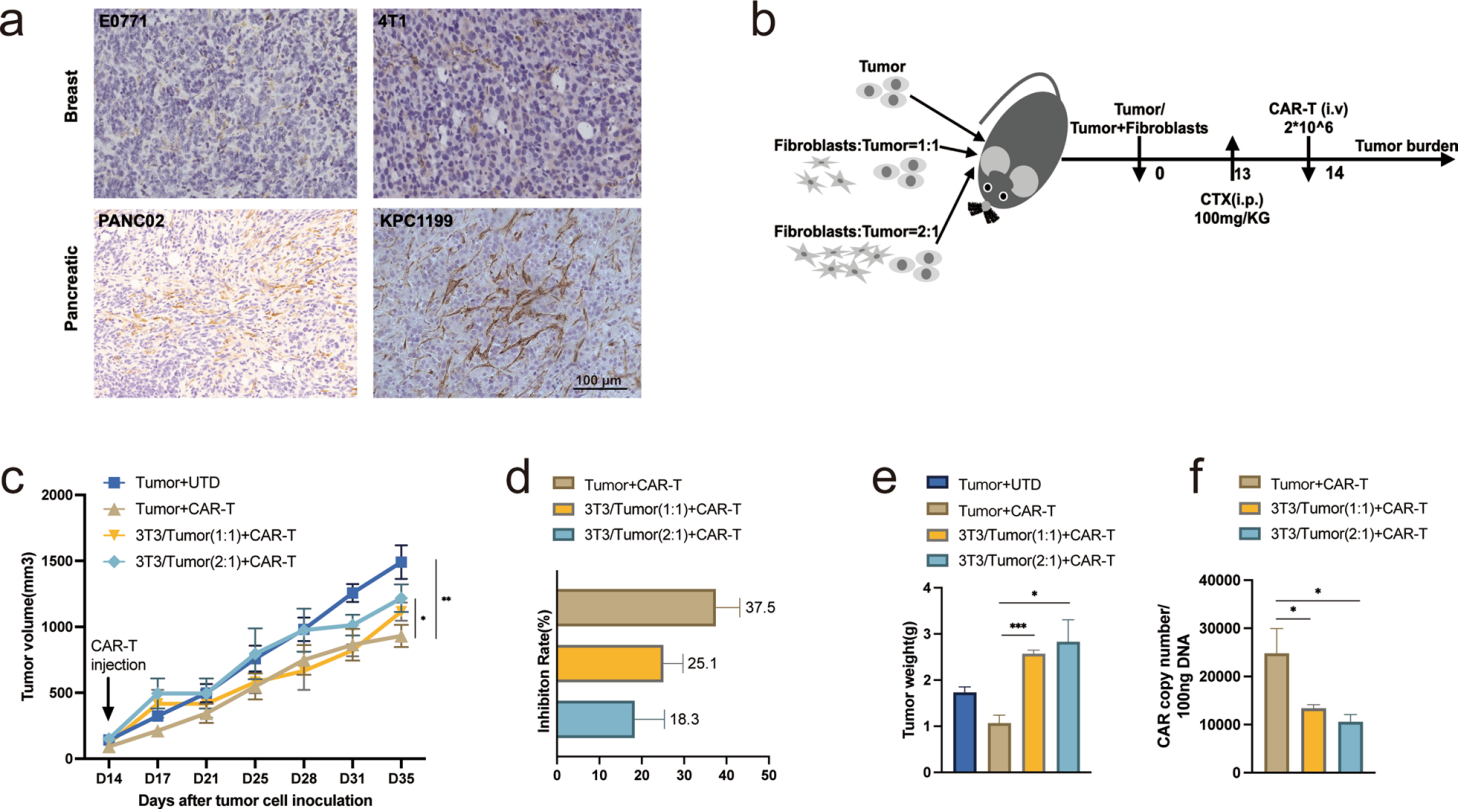
CAFs impair the antitumor activity of CAR-T cells in vivo. ** a** IHC staining of CAFs in 4T1 and E0771 breast and PANC02 and KPC1199 pancreatic tumor-bearing mice. **b**-**f** In vivo experimental of Antigen-Positive E0771 allografts. **b** Experimental scheme. Antigen-Positive E0771 tumor cells alone or combined with NIH 3T3 fibroblast were in situ inoculated into C57BL/6 mice, treated *i.p.* injection with CPA, and then given CAR-T cells (*i.v.*; n = 5 mice per group). **c** The tumor volume of tumors of each treatment group. **d** The tumor growth inhibition of each treatment group. **e** The tumor weight at the end point of the animal experiment. **f** CAR copy numbers in genomic DNA of residual tumors after therapy were measured by qRT-PCR (TaqMan probe). The images were obtained under original magnification 200×. Scale bars, 100 μm. All data are presented as the mean ± SEM of triplicate experiments. *p < 0.05, **p < 0.01, ***p < 0.001

Given that CAFs are the main cell types constituting pancreatic and breast cancer tumor stroma, this study examined CAFs expression in tumor tissue of 4T1 and E0771 breast as well as PANC02 and KPC1199 pancreatic tumor-bearing mice. By IHC, more CAFs were observed in the pancreatic tumor model than in the breast tumor model (Fig. 2a). We first explored the effect of CAFs on CAR-T cells antitumor activities in E0771 tumor cells syngeneic models with relatively low CAFs expression. As shown in Fig. 2b, mice bearing combinations of E0771-EGFR*v*III tumor cells and NIH 3T3 mouse fibroblasts in different ratios or E0771-EGFR*v*III tumor alone were treated by EGFR-targeted CAR-T cells. The results showed the transduction efficiency of EGFR-targeted CAR-T cells (Additional file 1: Fig.S2a). Interestingly, we found that allografts mixed with fibroblasts significantly reduce the activity of CAR-T cells, as shown by the lower tumor inhibition rate and heavier tumor weight (*p* < 0.05) (Fig. 2c-e). The body weight of mice showed no significant difference in all groups, suggesting no severe toxicity caused by the E0771 tumor, NIH 3T3, and CAR-T cells (Additional file 1: Fig. 2Sb). Mice were sacrificed at the end of the experiment, and genomic DNA was isolated from tumors to detect CAR copy numbers utilizing real-time quantitative PCR (RT-qPCR). The results showed that enhanced CAFs obviously decreased CAR-T copies in tumor tissues (*p* < 0.05) (Fig. 2f). These data suggested that CAFs could impair the efficacy of CAR-T therapy in the immunocompetent fibroblast-rich mouse model.

### Generation of FAP-targeted CAR-T and CLDN18.2-targeted CAR-T cells

**Fig. 3:**
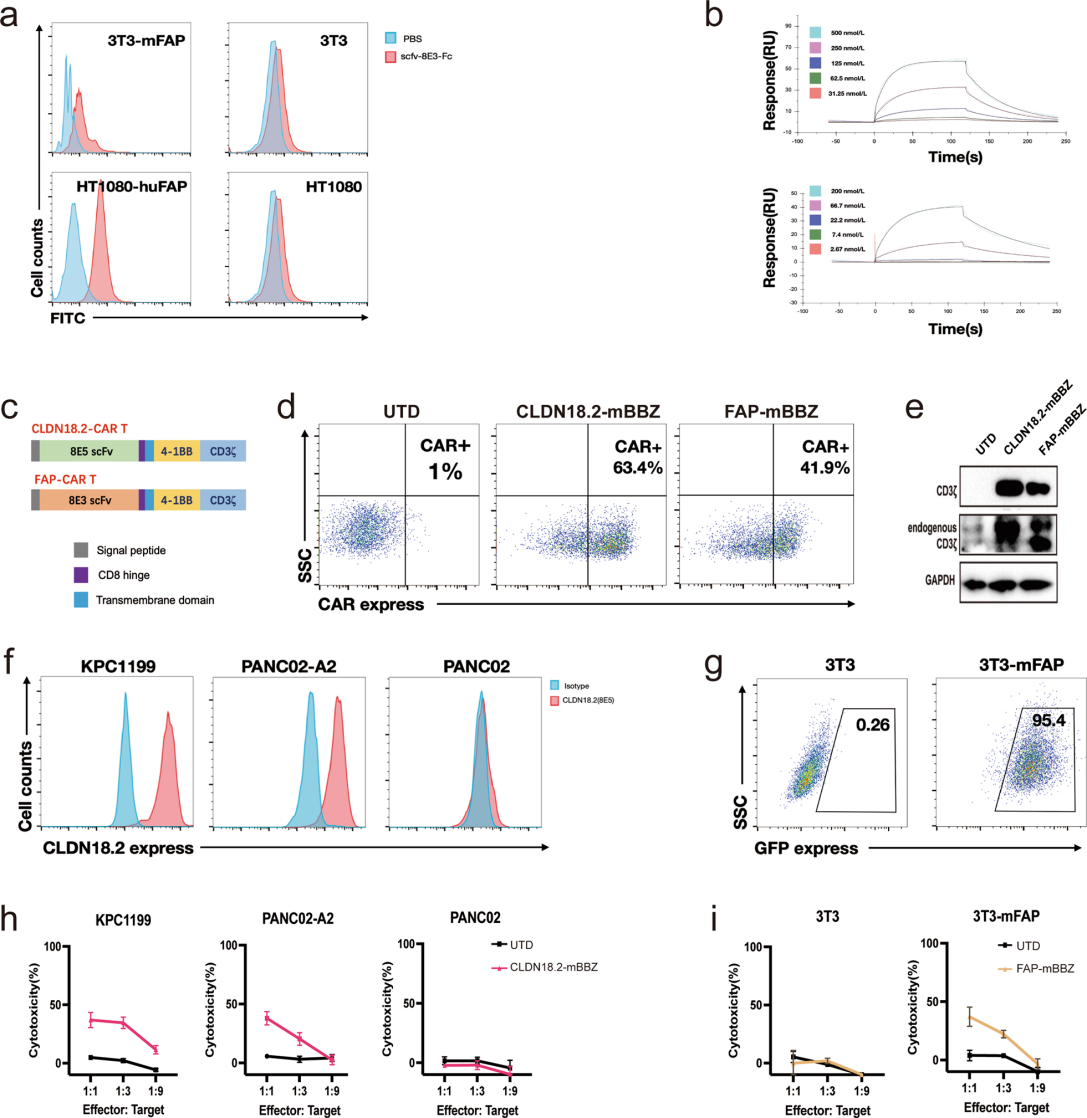
Generation of FAP-targeted CAR-T cells and CLDN18.2-targeted CAR-T cells. ** a** Binding specificity of the anti-FAP antibody to mFAP-transfected 3T3 or huFAP-transfected HT-1080. **b** Affinity measurements of anti-FAP antibody binding to FAP through BiacoreSurface Plasmon resonance (GE Healthcare). The upper shows its binding to mu-FAP and, the lower shows its binding to hu-FAP. **c** The construction of 8E3-mBBZ and 8E5-mBBZ CAR-T cells. This construct includes an extracellular antigen recognition region (8E5 targeting CLDN18.2 and 8E3 targeting FAP), a hinge, a transmembrane domain, an intracellular region of mouse 41-BB costimulatory molecules, and a mouse CD3-ζ chain. **d** The transduction efficiency of FAP-mBBZ and CLDN18.2-BBZ CAR-T CAR on splenic T cells derived from C57BL/6 was determined by flow cytometry. UTD cells served as negative controls. **e** Western blot of CD3-ζ in CAR-T. Glyceraldehyde 3-phosphate dehydrogenase (GAPDH) served as a loading control. **f** The expression of CLDN18.2 on KPC1199, PANC02, and PANC02-A2. Cells incubated with a mouse anti-mouse IgG antibody as a negative control. **g** The establishment of mFAP NIH 3T3 mouse fibroblast cells. NIH 3T3 was transduced by *p*WPT-GFP-mFAP lentivirus and further sorted by flow cytometry. **h**-**i** CAR-T cells were incubated with tumor or fibroblast cells at three effector: target (E: T) ratios for 18 h. Cell lysis was tested using a standard nonradioactive cytotoxicity assay. **h** Cytotoxic activities of CLDN18.2-mBBZ CAR-T cells on CLDN18.2-positive or CLDN18.2-negative tumor cells. **i** Cytotoxic activities of FAP-mBBZ CAR-T cells on FAP-positive or FAP-negative fibroblast cells

We first developed a novel anti-FAP antibody using phage display technology. The binding specificity of the anti-FAP antibody was tested on 3T3-mFAP (Flag) and HT1080-huFAP. The results in Fig. 3a indicated that 8E3-scFv bound specifically to FAP-expressing cells but not to cells without FAP expression. Additionally, the affinity measurements suggest a high binding affinity of 8E3-scFv to both human and murine FAP (Fig. 3b). Then, we constructed murine second-generation CAR 8E3-mBBZ, composed of an extracellular scFv derived from an 8E3 antibody linked through a hinge region to murine 4-1BB as well as CD3ζ intracellular signaling domains. Similarly, 8E5-mBBZ CAR was synthesized by CLDN18.2-specific scFv (hu8E5-2I) fused with murine 4-1BB and CD3ζ intracellular signaling domains (Fig. 3c) [4]. Murine T cells were retrovirally transduced to generate CAR-T cells, and the transduction efficiency of 41% and 62% in 8E3-mBBZ CAR-T (FAP-targeted CAR-T, FAP-mBBZ) and 8E5-mBBZ CAR-T (CLDN18.2-targeted CAR-T, CLDN18.2-mBBZ) cells were detected by flow cytometry, respectively (Fig. 3d). Given that FAP was expressed at low levels in mouse fibroblast NIH 3T3, mFAP-positive NIH 3T3 was transduction by lentivirus to stably express murine FAP (Fig. 3g). CLDN18.2-positive PANC02, KPC1199, and mFAP-positive NIH 3T3 cells were sorted out successfully by flow cytometer (Fig. 3f, g). To determine the function of FAP-mBBZ CAR-T cells in vitro, cytotoxicity assays were performed. As shown in Fig. 3h, the specific lysis result of the CLDN18.2-positive PANC02 and KPC1199 cells indicates the cytotoxic activity of CLDN18.2-mBBZ CAR-T cells to CLDN18.2-positive cells. Besides, FAP-mBBZ CAR-T cells efficiently lyse mFAP-positive NIH 3T3 cells, but almost no lysis was observed in NIH 3T3 without FAP expression (Fig. 3i). UTD T cells did not lyse the target cells tested. These data suggest that FAP-mBBZ CAR-T cells specifically recognize and attack mFAP-positive cells in vitro.

### Sequential therapy of FAP-targeted and CLDN18.2-targeted CAR-T cells dramatically improved antitumor efficacy against CLDN18.2-Positive tumor-bearing mice

**Fig. 4:**
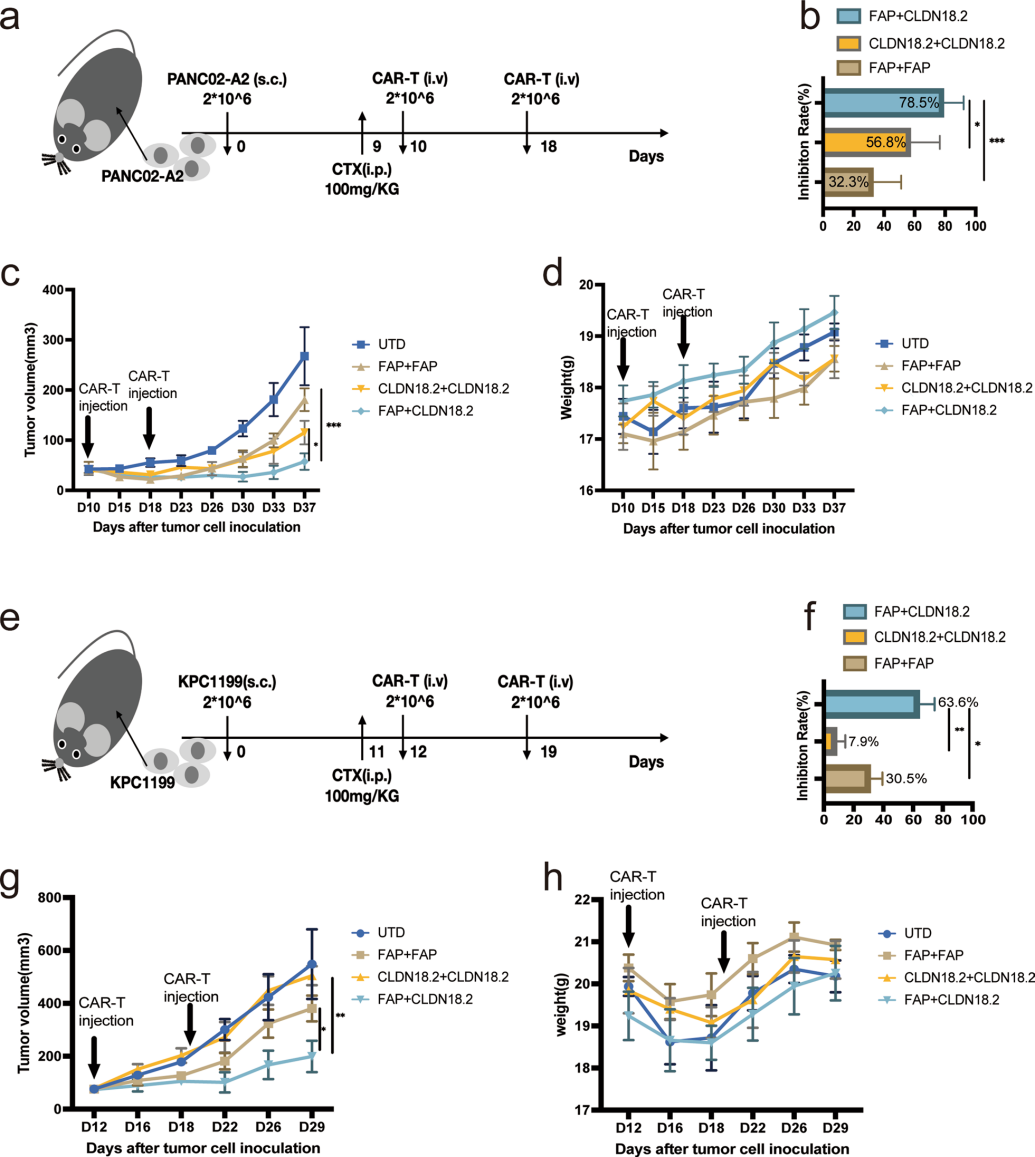
Sequential therapy of FAP-targeted and CLDN18.2-targeted cells dramatically improved antitumor efficacy against CLDN18.2-positive tumor-bearing mice. **a**-**d** In vivo experimental design. C57BL/6 mice were injected *s.c.* with PANC02-A2 cells and allowed to establish for 10 days. Mice were assigned to four experimental groups. The first and second CAR-T cells were injected on day 10 and day 18, respectively (*i.v.*; n = 5 mice per group). **b** The tumor growth inhibition of each treatment group. **c** The volume of tumors of each treatment group. **d** The body weight of mice of each treatment group. **e**-**h** In vivo experimental design. C57BL/6 mice were injected *s.c.* with KPC1199 cells and allowed to establish for 12 days. Mice were assigned to four experimental groups. The first and second CAR-T cells were injected on day 12 and day 19, respectively (*i.v.*; n = 5 mice per group). **f** The tumor growth inhibition of each treatment group. **g** The volume of tumors of each treatment group. **h** The body weight of mice of each treatment group. All data are presented as the mean ± SEM of triplicate experiments. *p < 0.05, **p < 0.01, ***p < 0.001

To begin with, we used immunocompetent mice bearing CLDN18.2-positive PANC02 allograft tumor to explore the antitumor activities of FAP-mBBZ CAR-T cells in vivo (Additional file 1: Fig. S3a). As shown in Additional file 1: Fig.S3b, 7 days after CAR-T injection, the FAP-mBBZ CAR-T cells significantly suppressed the growth of allograft tumor (*p* < 0.05) and lasted for about one week. The mixture of FAP-mBBZ and CLDN18.2-mBBZ CAR-T cells could not show superior antitumor activities to mono-CAR-T. Besides, similar to other groups, no significant body weight loss was in mice treated with the FAP-mBBZ CAR-T cells (Additional file 1: Fig. S3c). A significant decrease of CAR copies in tumor tissue was found 7 days compared with 14 days after T cell infusion in the FAP-mBBZ CAR-T treated group (Additional file 1: Fig. S3d). Similar phenomena were found in CLDN18.2-mBBZ cells and the mixture CAR-T cells treated groups. Because the FAP-mBBZ CAR-T cells displayed a preliminary antitumor effect on day 7 and showed more significant infiltration of CAR-T cells on this day than 14 days after administration, 7 days were selected for the interval time of sequential therapy. Then we further explore the antitumor activities of FAP-mBBZ and CLDN18.2-mBBZ CAR-T cells sequential treatment in vivo. Allograft tumors were established with CLDN18.2-positive PANC02-A2 cells in immunocompetent mice. Mice were then assigned to four experimental groups, including FAP + CLDN18.2, FAP + FAP, CLDN18.2 + CLDN18.2, and UTD. Those in the sequential group (FAP + CLDN18.2) received the prior FAP-mBBZ CAR-T cells on day 10, and CLDN18.2-mBBZ CAR-T cells were subsequently infused on day 18 after tumor cell inoculation (Fig. 4a). At these two time-points, mice in the FAP + FAP, CLDN18.2 + CLDN18.2, and UTD groups were repeatedly infused with FAP-mBBZ CAR-T, CLDN18.2-mBBZ CAR-T and UTD T cells, respectively. All three CAR-T treated groups had an antitumor effect, while the efficacy of the FAP + FAP group was the weakest (Fig. 4b-c). On day 37, the tumors in the sequential treatment group were significantly smaller than those in the CLDN18.2 + CLDN18.2 group (*p* < 0.05) as well as those in the FAP + FAP group (*p* < 0.001) (Fig. 4b, c). Furthermore, the antitumor activities of sequential treatment were also tested in mice bearing established KPC1199 tumor allografts (Fig. 4e). The observation stopped because fester occurred in one mouse on day 29 after tumor cell inoculation. Sequential treatment inhibited the growth of the tumor more than those in the FAP + FAP group (*p* < 0.05), as well as those in the CLDN18.2 + CLDN18.2 group (*p* < 0.01) (Fig. 4f–g). Notably, FAP-mBBZ CAR-T cells had better antitumor activities than CLDN18.2-mBBZ CAR-T cells in the KPC1199 tumor model (*p* < 0.05), which was contrary to the PANC02 tumor model (Fig. 4b–c). Meanwhile, in both PANC02 and KPC1199 models, during all therapeutic processes, the groups had no significant differences in body weights (Fig. 4d and h). No morphological abnormality appeared in the H&E staining of the heart, liver, spleen, lung, and kidney tissue sections at the end of therapy (Additional file 1: Figs. S4, S5). Thus, we did not find severe toxicity in mice treated with FAP-mBBZ and CLDN18.2-mBBZ CAR-T cells. These results suggested that the sequential treatment of FAP-mBBZ and CLDN18.2-mBBZ CAR-T cells had better therapeutic potential than the same CAR-T cells in CLDN18.2-positive tumor-bearing mice.

### Sequential therapy of FAP-targeted and CLDN18.2-targeted CAR-T cells significantly increase the accumulation of CD8 ^+^ T and CAR-T cells

**Fig. 5:**
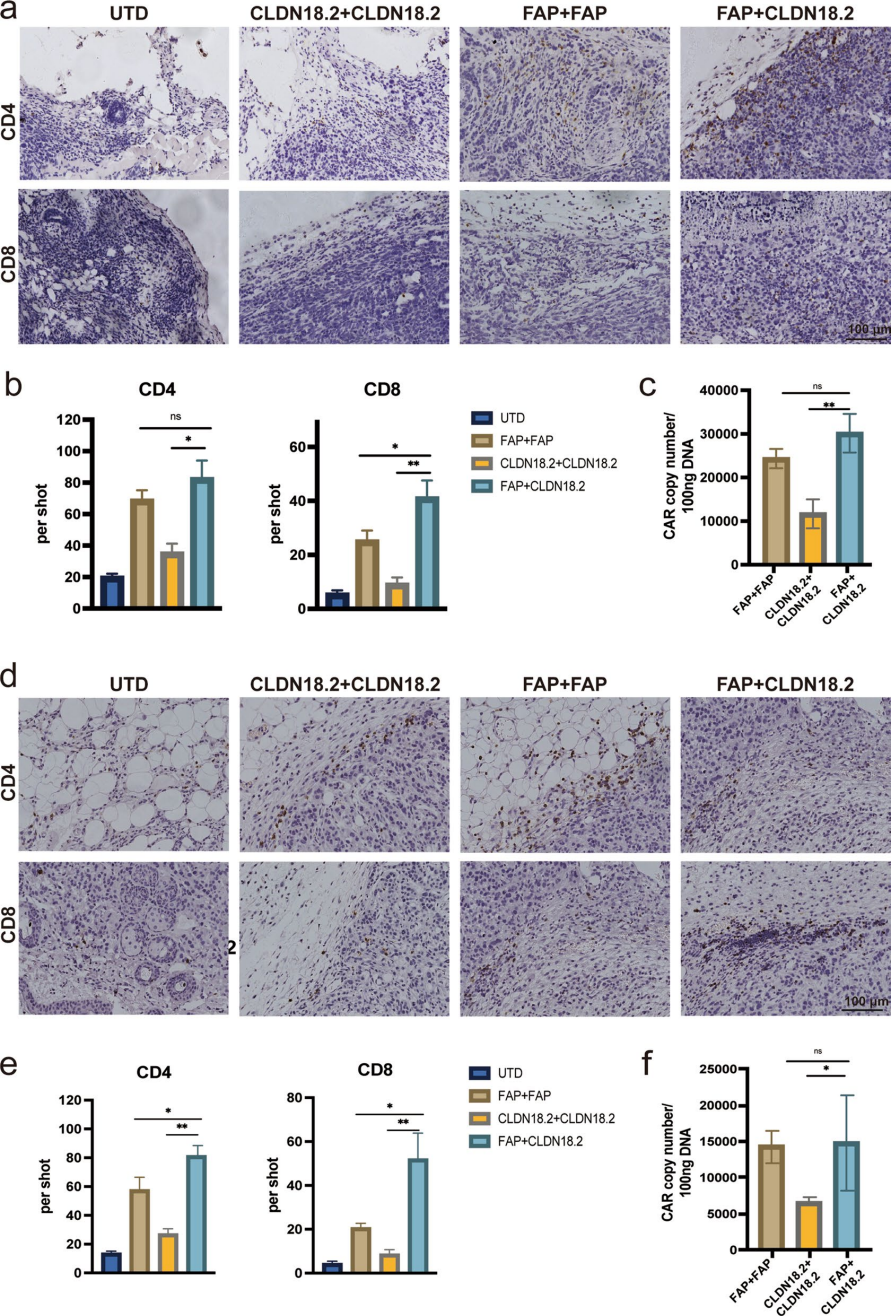
Sequential therapy of FAP-targeted and CLDN18.2-targeted CAR-T cells dramatically increase the accumulation of CD8^+^ T and CAR-T cells. **a–c** Tumor tissues were harvested from the mice at the end of therapy in the PANC02 model. **a** The representative images of tumor-infiltrating T cells immunostained with anti-CD4 and anti-CD8 from each treatment group. **b** Histograms show the quantification of T cells in tumor tissues. **c** CAR copy numbers in genomic DNA of residual tumors from each treatment group were measured by qRT-PCR (TaqMan probe). **d**–**f** Tumor tissues were harvested from the mice at the end of therapy in the KPC1199 model. **d** The representative images of tumor-infiltrating T cells immunostained with anti-CD4 and anti-CD8 from each treatment group. **e** Histograms show the quantification of T cells in tumor tissues. **f** CAR copy numbers in genomic DNA of residual tumors from each treatment group were measured by qRT-PCR (TaqMan probe). The images were obtained under original magnification 200×. Scale bars, 100 μm. All data are presented as the mean ± SEM of triplicate experiments. *p < 0.05, **p < 0.01

To elucidate the mechanism of the enhanced antitumor ability of FAP-mBBZ and CLDN18.2-mBBZ CAR-T cells sequential treatment, we harvested PANC02 allografts and examined the infiltration of T cells. Compared with the FAP + FAP and CLDN18.2 + CLDN18.2 groups, increased infiltration of CD8^+^ T was observed in the sequential treatment group by IHC staining (*p* < 0.05) (Fig. 5a-b). Besides, more CD4^+^ T infiltration was found in the sequential and FAP + FAP groups than in the CLDN18.2 + CLDN18.2 group (*p* < 0.05) (Fig. 5a-b). DNA CAR copy numbers were examined to represent CAR expression in tumor tissue, which was more significantly elevated in the sequential treatment group than in the CLDN18.2 + CLDN18.2 group (*p* < 0.05) (Fig. 5c). Similar results were found in the KPC1199 model. More CD8^+^ and CD4^+^ T cells were observed in the sequential treatment group than in other groups by IHC staining (*p* < 0.05) (Fig. 5d-e). Additionally, the sequential treatment led to increased CAR copies in tumor tissues than twice treatment of CLDN18.2-mBBZ CAR-T cells (*p* < 0.05) (Fig. 5f). These data suggested that sequential treatment could improve the accumulation of CAR-T cells in the tumor site.

### FAP-targeted CAR-T cells eliminate CAFsin vivo

**Fig. 6:**
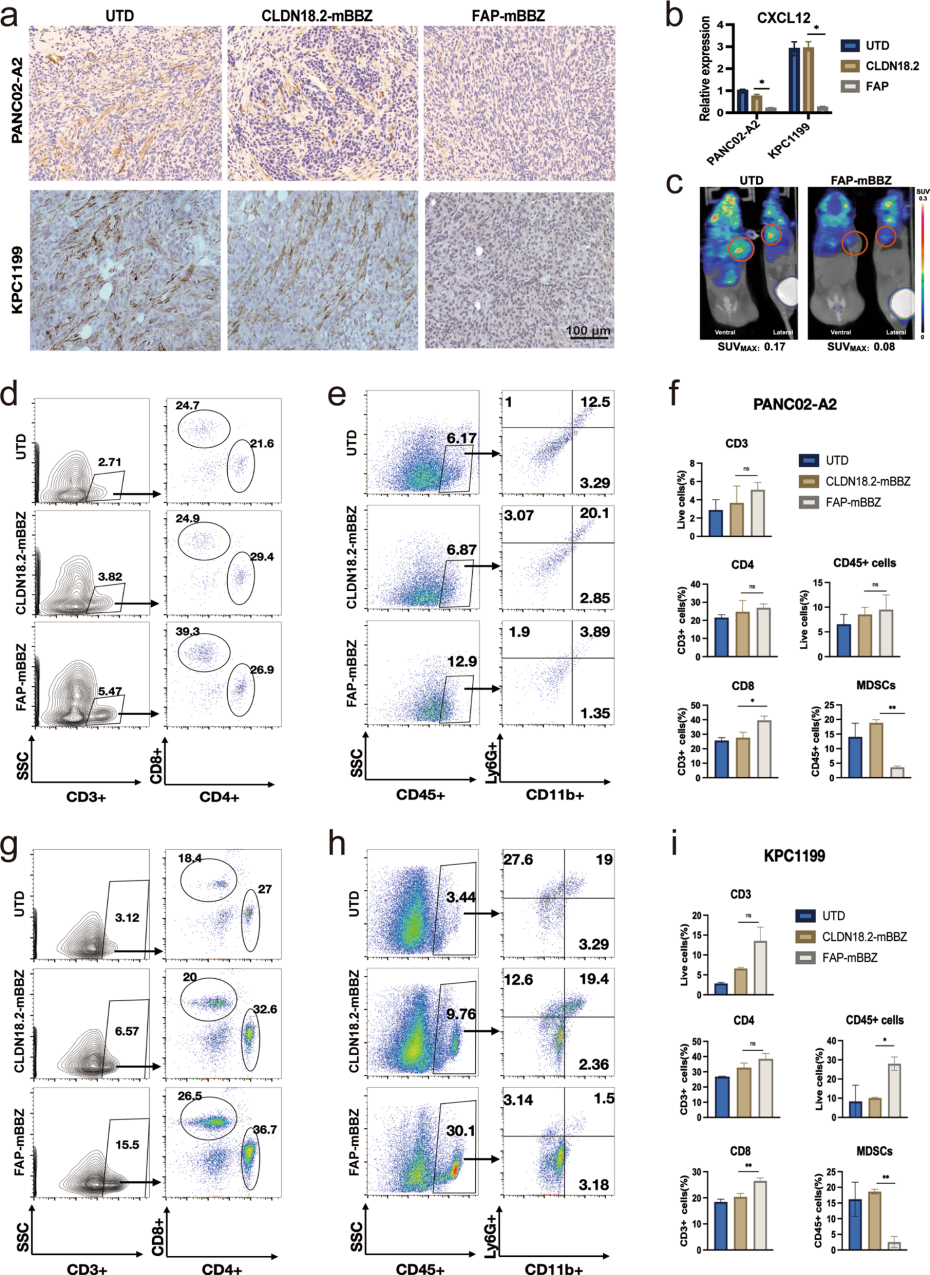
FAP-targeted CAR-T cells eliminate CAFs and change tumor microenvironment in vivo. C57BL/6 mice were injected *s.c.* with PANC02-A2 or KPC1199 cells and treated with CAR-T cells after the tumor was established. To exclude the influence of tumor volume difference by distinct CAR-T cells, tumor tissue was harvested 7 days after treatment. **a–c** Analysis of the CAFs in tumor tissues of PANC02-A2 and KPC1199 allografts. **a** The representative images of CAFs immunostained with α-SMA in tumor tissues from each treatment group. **b** q-PCR measured the mRNA expression of CXCL12 in tumor tissues from each treatment group. **c** PET image labeling FAP by FAPI in PANC02-A2 allografts 7 days after the treatment of FAP-mBBZ or UTD T cells.  **d–f**  Analysis of the immune cell in tumor tissues of PANC02-A2 allografts. **d** The representative flow cytometry plots showing the frequencies of tumor-infiltrating CD8^+^ and CD4^+^ cells in CD3^+^ T of each treatment group. **e** The representative flow cytometry plots showing the frequencies of tumor-infiltrating CD45^+^ immune cells and MDSCs of each treatment group. **f** Flow cytometry quantitation of tumor-infiltrating immune cells of each treatment group. **g–i** Analysis of the immune cell in tumor tissues of KPC1199 allografts. **g** The representative flow cytometry plots showing the frequencies of tumor-infiltrating CD8^+^ and CD4^+^ cells in CD3^+^ T of each treatment group. **h** The representative flow cytometry plots showing the frequencies of tumor-infiltrating CD45^+^ immune cells and MDSCs of each treatment group. **i** Flow cytometry quantitation of tumor-infiltrating immune cells of each treatment group. The images were obtained under original magnification 200×. Scale bars, 100 μm. All data are presented as the mean ± SEM of triplicate experiments. *p < 0.05, **p < 0.01, *** *p* < 0.001

With the successful selective attack on FAP-positive fibroblast in vitro, we evaluated whether FAP-mBBZ CAR-T could eliminate CAFs in vivo. The mice were administered FAP-mBBZ or CLDN18.2-mBBZ CAR-T cells when tumors were established. Mice were sacrificed and harvested tumor tissue 7 days after the treatment to exclude the influence of tumor volume difference by distinct CAR-T cells. A dramatic decrease in the CAFs measured by immunostaining was observed in the FAP-mBBZ group, while CAFs were abundant in tumor lesions of the CLDN18.2-mBBZ CAR-T and UTD groups (Fig. 6a). In addition, much lower level of mRNA expressions of CXCL12 which mainly produced by CAFs in the tumor site was observed in the FAP-mBBZ CAR-T group than those from CLDN18.2-mBBZ CAR-T and UTD groups (Fig. 6b). To determine further the effect of FAP-mBBZ CAR-T cells on CAFs, PET imaging using FAP inhibitor (FAPI) probes to label FAP metabolism was conducted 7 days after CAR-T treatment [31]. The high uptake of FAPI revealed a large number of CAFs at the site of the tumor in the UTD group. However, a dramatic decrease in FAPI uptake was observed in the FAP-mBBZ CAR-T group, suggesting it could eliminate CAFs effectively (Fig. 6c).

### FAP-targeted CAR-T cells increase the infiltration of cytotoxic T cells and decrease MDSCs recruitment in tumor

The phenotype of T cells in tumor tissue was tested via flow cytometry to evaluate the potential effect of FAP-mBBZ CAR-T cells on the infiltration of T cells. Compared with the CLDN18.2-mBBZ CAR-T group, the FAP-mBBZ CAR-T group showed more CD8^+^ T cells in tumor tissue, whereas the CD4^+^ T cells in each group showed no differences (Fig. 6d, g). Besides, no significant differences appeared in the exhaustion markers of T cells (PD1, TIM3, and LAG3) and PD-L1 of tumor cells in the tumor site after CLDN18.2-mBBZ or FAP-mBBZ CAR-T treatment (Additional file 1: Fig.S6b, d). These data suggested that FAP-mBBZ CAR-T cells could improve the infiltration and antitumor efficiency of CAR-T cells in both pancreas cancer mouse models. To further explore the influence of FAP-mBBZ CAR-T cells in the immune microenvironment, we analyzed the infiltration of immune cells, including MDSCs, macrophages, DCs, and NKs, via flow cytometry seven days after CAR-T treatment. Large amounts of MDSCs were detected in CLDN18.2-positive tumor-bearing mice tumor tissue. After the FAP-mBBZ CAR-T treatment, the ratio of MDSCs among all immune cells in tumor tissue was significantly decreased in PANC02-A2 models (Fig. 6e–f). However, no significant differences appeared in this ratio after the CLDN18.2-mBBZ CAR-T treatment (Fig. 6e–f). Similar trends regarding the ratio of MDSCs were also observed in KPC1199 models (Fig. 6h, i). A higher FAP expression and CAFs characteristics were found to be significantly associated with the more active MDSCs characteristics in TCGA-PDAC patients in further bioinformatics analysis (Additional file 1: Fig.S6e–f). Besides, the infiltration of CD45^+^ immune cells was increased after FAP-mBBZ CAR-T treatment in the KPC1199 model, which remains generally stable in the PANC02-A2 model (Fig. 6e, h). In addition, no significant difference in the ratio of macrophages, DCs, and NKs between the CLDN18.2-mBBZ and FAP-mBBZ CAR-T groups was observed. (Additional file 1: Fig. S6a, c).

## Discussion

**Fig. 7:**
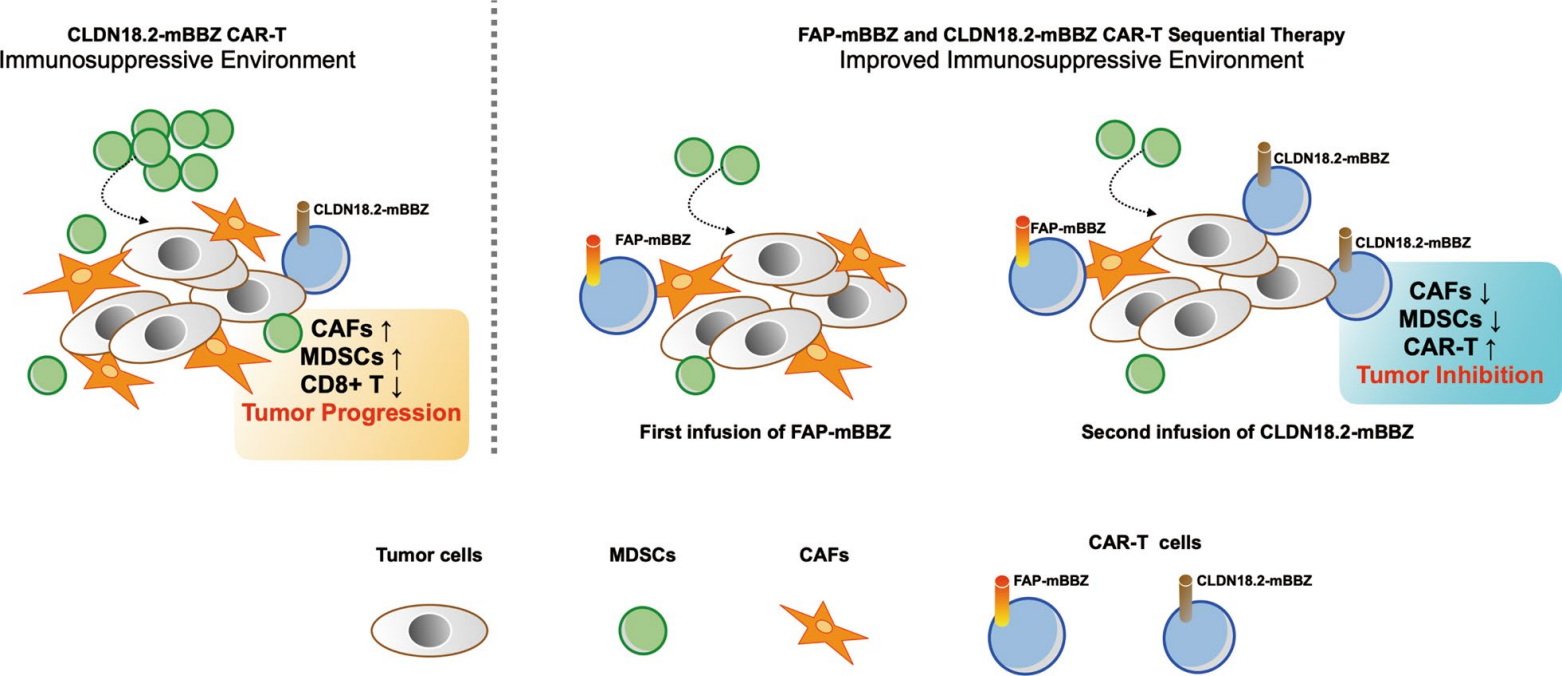
Schema: Sequential Therapy enhances the antitumor ability of CAR-T by suppressing the infiltration of MDSCs. Left: CAFs are crucial in TME, forming an immune surveillance barrier and perpetuating tumor-promoting. The CAFs could also promote recruit monocytes from the bone marrow, such as MDSCs, to form a tumor-suppressing microenvironment, suppressing CAR-T cell function. Right: FAP-targeted CAR-T cells eliminate CAFs via specific recognition of FAP on CAFs in the tumor microenvironment. Further, CLDN18.2-targeted CAR-T cells could also increase cytotoxic T cells and inhibit the recruiting of MDSCs. With an improved immune suppressive microenvironment, the antitumor effect of the sequential infusion of CLDN18.2-targeted CAR-T cells were enhanced

CAR-T cells have encountered significant impediments in the treatment of solid tumors. TME is one of the main issues which form a barrier to immune surveillance and exerts immunosuppressive features [6, 10]. Several corresponding strategies were proposed, and specific cell components of the TME have emerged as promising targets for CAR-T therapy [32]. For example, one recent CAR-T preclinical study targeting tumor necrosis factor-related apoptosis-inducing ligand receptor 2 (TR2) expressed on MDSCs reshaped the immunosuppressive TME successfully and exhibited superior antitumor efficiency in solid tumor model [32, 33]. To improve the poor accumulation of CLDN18.2-targeted CAR-T cells in pancreatic cancer revealed in our previous study [7], we developed one kind of novel FAP-targeted CAR-T cells. Sequential treatment triggers a solid antitumor immune response and improves therapeutic efficacy in two murine pancreatic cancer models, at least partially through inhibiting MDSCs recruitment (Fig. 7).

CAFs are one of the most prominent and active components in the TME of PDAC [8]. Multiple studies have shown that CAFs create a hostile TME for tumor immune evading, including attack from cytotoxic T cells [8, 12, 14]. In this study, artificially mixing fibroblast cells with tumor cells impaired the infiltration and antitumor effect of CAR-T cells in vivo, indicating CAFs in TME as negative modulators for CAR-T cells efficacy and providing a rationale for reshaping immunosuppressive microenvironment by eliminating CAFs. FAP overexpression in CAFs has been consistently reported and associated with poor prognosis [15, 16, 34]. Our bioinformatics analysis further revealed that FAP expression levels were highly correlated with various immunosuppressive features and EMT in pancreatic patients. Different FAP-targeted strategies are being explored. For instance, FAP-targeted monoclonal antibody conjugated to maytansinoid and vaccine was developed and demonstrated antitumor efficacy by destroying TME vessels and fibroblast [17–19]. FAP-targeted CAR-T cells were also developed to control tumor growth by activating the function of endogenous immune cells [21, 22]. To minimize the immunogenicity of CAR-T cells, we develop fully-human FAP-targeted CAR-T cells and provide evidence that it attack mouse FAP-positive cells effectively. Although some FAP-positive cells are present in the placenta and uterine stroma, it is not or at low levels expressed in healthy adult tissues and seems a theoretically safe target for CAR-T therapy [16]. However, one previous study revealed that FAP-targeted CAR-T cells induced lethal ototoxicity and cachexia by killing pluripotent stem cells in the bone marrow stroma, which aroused great concern [20]. Further study found it may be partially due to the CAR constitution and therapeutic scenarios, including whole-body irradiation [22]. Besides, several following studies demonstrated the safety and effectiveness of FAP-targeted CAR-T cells constructed by different scFv antibodies [20, 22]. Our study detected neither obvious damage to vital organs nor systemic toxicity after treatment, indicating that the FAP-targeted CAR-T cells developed here should be safe and tolerable.

Previous studies report that FAP-targeted CAR-T cells decreased FAP-positive stromal cells in mouse mesothelioma and lung models [21, 22]. Similarly, in our study, several lines of evidence, including IHC, FAP-labeled PET-CT, and RT-qPCR, revealed that most CAFs were depleted after treatment of FAP-targeted CAR-T cells, which were not seen in CLDN18.2-targeted CAR-T cells. The duration of elimination was at least one week. Although FAP-targeted CAR-T cells eliminate CAFs effectively, its antitumor effect remains to be further improved in animal experiments. Therefore, based on the following considerations, we have established FAP-targeted and CLDN18.2-targeted CAR-T cells sequential therapy, aiming to improve the therapeutic efficacy of PDAC. To begin with, due to the heterogeneity of tumor cells, it is found that antigen loss or downregulation may cause tumor recurrence after CAR-T therapy [10]. CAFs, as stromal cells, are more genetically stable than tumor cells [32]. Moreover, several studies found that the CAR construct can trigger anti-CAR immune responses, affecting CAR-T therapy efficiency. For example, the effect of repeated CD19-targeted CAR-T cells infusion was not satisfactory, partly due to endogenous T cells mediated anti-CAR response [35]. Another study of CD19-targeted CAR-T cells with humanized scFv revealed that anti-drug antibodies (ADA) were correlated with the disease relapse risk [36]. Given that scFv is an essential non-self constitution in CAR, for the sake of reducing immunogenicity, FAP-targeted and CLDN18.2-targeted CAR-T cells were subsequently infused instead of repeated infusion the mixture of those two CAR-T. Furthermore, in the clinical stage, sequential CAR-T treatment has been explored in hematology. One clinical trial (ChiCTR1800014457) recently revealed prolonged and significant efficiency and safety of sequential CD19-CD22-CD20 CAR-T therapy in pediatric patients suffering relapsed/refractory Burkitt lymphoma (r/r BL), even in patients with central nervous system (CNS) involvement [37, 38]. Further studies of this group showed that short-interval sequential infusion of different CAR-T cells enhanced its cell expansion and the antitumor effect [39]. The interval time of sequential treatment was settled as 7 days, based on the accumulation of infiltrated CAR-T cells and the onset time of FAP-targeted CAR-T treatment revealed in our in vivo study. In addition, prior CAR-T cells are likely to be exhausted due to the long-interval time, thus reducing the efficiency [39].

We first investigated whether infiltrated T cells changed in vivo to explore how FAP-targeted CAR-T cells enhance subsequent CLDN18.2 targeted CAR-T therapy. 7 days after FAP-targeted CAR-T cells infusion, we observed significant CD8^+^ T as well as CAR-T cells accumulation at the tumor site in both the PANC02-A2 and KPC1199 models. Similarly, a previous report also showed TNF-α^+^CD4^+^ T cells and CD8^+^ T cells increased after FAP-targeted CAR-T cells infusion [22]. Besides, the same study found more IFN-γ^+^ CD8^+^ T cells. It is well known that IFN-γ production can induce PD-L1 expression [40]. However, no apparent changes in tumor PD-L1 and T cells exhausted markers were observed in our study. Briefly, FAP-targeted CAR-T cells remodel the hostile TME of the PDAC model and provide a favorable environment for subsequent CAR-T therapy.

Notably, we observed a significant decrease in the number of MDSCs in tumor tissue of the FAP-targeted CAR-T treatment group compared with the CLDN18.2-targeted CAR-T and UTD groups. Given the dominant immunosuppressive role of MDSCs, the elimination of MDSCs by FAP-targeted CAR-T therapy should contribute to the increased efficacy of the sequential treatment. Formed by a heterogeneous group of immature myeloid cells, MDSCs dampen the proliferation and activation of cytotoxic T cells [41]. This effect has also been found in clinical studies where patients with a lower presence of MDSC are more likely to respond to T-cell-based immune checkpoint blockade (ICB) [42, 43]. Mechanically, MDSCs suppress T cell functions mainly via induction of reactive oxygen species (ROS) and PD-L1 as well as depletion of essential nutrients [41, 44]. Importantly, our group previously found that MDSCs could induce CD8^+^ T cell apoptosis and impair the proliferation and efficacy of CAR-T cells in murine models, including breast and colon cancer [45, 46]. Besides, CAFs, major sources of chemokines, including CXCL12, CCL2 and CXCL1, can recruit MDSCs to tumor sites and promote their differentiation and activation [47, 48]. Consistently, the mRNA expression level of CXCL12 was significantly decreased after FAP-targeted CAR-T cells infusion in our in vivo study. One recent report further demonstrated a significant spatial interaction between CAFs and monocytic myeloid cells in the TME by imaging mass cytometry [48]. Similarly, our bioinformatic analysis revealed the correlation between FAP expression and MDSCs in pancreatic cancer patients in the TCGA database, further proving the crosstalk of those two types of cells in TME. Thus, we speculated that MDSCs recruitment, differentiation, and immunosuppressive effect were reduced due to CAFs clearance by FAP-targeted CAR-T cells, then improving the existing CD8^+^ T cells. Interestingly, we discovered that FAP-targeted CAR-T cells inhibit MDSCs in tumor sites and further promote the infiltration and survival of sequentially CLDN18.2-targeted CAR-T cells.

FAP-targeted CAR-T cells inhibited tumor growth for a short time in both PDAC models, even though the second infusion was administrated. As previously found, CAFs promote tumor growth, invasion, and progress, partly explaining that tumor growth was controlled initially [12]. However, with the elimination of CAFs in TME, the antitumor effect of FAP-targeted CAR-T cells against tumor cells is limited; thus, the tumors grow resistant to the treatment. In addition, several studies have revealed the bystander effect of CAR-T therapy in the clearance of antigen-negative tumor cells, which kill tumors based on the secretion of IFN-γ and the Fas/FasL pathway [49, 50]. Thus, FAP-targeted CAR-T cells are likely to attack CLDN18.2-positive tumor cells at the initial stage of treatment in our animal experiment by this mechanism.

Nevertheless, there were still some limitations in our study. Further investigations will be needed. First, we did not use orthotopic tumor models to investigate the antitumor efficacy and toxicity of CAR-T cells in vivo. Even though we observed abundant CAFs in tumor sites, orthotopic studies are needed in the future. Secondly, although repeated infusion may increase the risk of ADA, the antitumor effect of dual-antigen CAR-T cells and the mixture of two different CAR-T cells need further exploration. Thirdly, it is necessary to do more work to explore the optimal sequential treatment dose combinations and interval time, particularly based on CAR-T cells and CAFs dynamic change with real-time tracing technology, before translating into clinical application.

Together, in this study, we identified the potent antitumor effects of FAP-targeted and CLDN18.2-targeted CAR-T cells sequential therapy in murine pancreatic cancer models at least partially through the improved accumulation of CAR-T cells and reduced recruitments of MDSCs to the tumor sites. In summary, our findings suggest a promising strategy to improve further the CLDN18.2 CAR-T cells for treating pancreatic cancer.

## Supplementary Information

**Additional file 1: Figure S1.** (a) Box plot comparing fibroblast characteristics between high-FAP *vs.* low-FAP groups estimated by IOBR. (b) Heatmap showing fibroblast characteristics between high-FAP *vs.* low-FAP groups estimated by IOBR. (c) Pan-cancer analysis of FAP protein expression by IHC staining. **Figure S2.** (a) The transduction efficiency of 806-28Z CAR on splenic T cells derived from C57BL/6 was determined by flow cytometry. UTD cells served as negative controls. (b) The body weight of mice of each treatment group. All data are presented as the mean ± SEM of triplicate experiments. **Figure S3.** (a-d) In vivo experimental design. C57BL/6 mice were injected *s.c.* with PANC02-A2 cells and allowed to establish for 10 days. Mice were assigned to four experimental groups. Then, CAR-T cells were injected on day 10 (*i.v.*; n = 5 mice per group). Additional three mice in each group were used for harvesting tumor tissue during and after the CAR-T treatment. (b) The volume of tumors of each treatment group. (c) The body weight of mice of each treatment group. (d) CAR copy numbers in genomic DNA of residual tumors from each treatment group were measured by qRT-PCR (TaqMan probe). All data are presented as the mean ±SEM of triplicate experiments. *p < 0.05. **Figure S4.** Hematoxylin and eosin (HE) staining of important organs. Heart, liver, spleen, lung, and kidney were analyzed by HE staining. After study termination, the specimens were harvested from PANC02-bearing mice. The images were obtained under 200×magnification. The scale bar was 100 μm. The data shown are representative of experiments with similar results. **Figure S5.** Hematoxylin and eosin (HE) staining of important organs. Heart, liver, spleen, lung, and kidney were analyzed by HE staining. After study termination, the specimens were harvested from KPC1199-bearing mice. The images were obtained under 200×magnification. The scale bar was 100 μm. The data shown are representative of experiments with similar results. **Figure S6.** (a-b) Analysis of the immune cell in tumor tissues of PANC02-A2 allografts. (a) Quantitation of tumor-infiltrating immune cells of each treatment group by flowcytometry. (b) Surface expression of exhaustion marker in T cells and PD-L1 in tumor cells of each treatment group determined by flow cytometry. (c-d) Analysis of the immune cell in tumor tissues of KPC1199 allografts. (c) Quantitation of tumor-infiltrating immune cells of each treatment group by flow cytometry. (d) Surface expression of exhaustion marker in T cells and PD-L1 in tumor cells of each treatment group determined by flow cytometry. (e) Correlation between FAP expression and MDSCs characteristics estimated by IOBR in TCGA-PDAC patients. (f) Correlation between CAFs and MDSCs characteristics estimated by IOBR in TCGA-PDAC patients. (g) Gating strategies for flow cytometry. All data are presented as the mean ± SEM of triplicate experiments.

## Data Availability

The data that support the findings of this study are available from the corresponding author upon reasonable request. Publicly available datasets were analyzed in the bioinformatics part of this study. These data can be found here: cBioPortal for Cancer Genomics: https://www.cbioportal.org/; Human Protein Atlas: https://www.proteinatlas.org/.

## Abbreviations

CAFs: Cancer-associated fibroblasts
CAR: Chimeric antigen receptors
CLDN18.2: Claudin18.2
FAP: Fibroblast activation protein
GSEA: Gene Set Enrichment Analysis
HPA: Human Protein Atlas
IHC: Immunohistochemistry
IOBR: Immune Oncology Biological Research
MDSCs: Myeloid-derived suppressor cells
PDAC: Pancreatic ductal adenocarcinoma
ssGSEA: Single Sample Gene Set Enrichment Analysis
SUV: Standardized uptake value
TAM: Tumor-associated macrophage
TCGA: The Cancer Genome Atlas
Treg: Regulatory T cells
UTD: Untransduced
