# Supplementary material for: FAP-targeted CAR-T suppresses MDSCs recruitment to improve the antitumor efficacy of claudin18.2-targeted CAR-T against pancreatic cancer
Source: J Transl Med. 2023 Apr 12;21:255. doi: 10.1186/s12967-023-04080-z (PMC10091631; doi:10.1186/s12967-023-04080-z)
Supplement: Supplementary file 1 — Additional file 1: Figure S1. (a) Box plot comparing fibroblast characteristics between high-FAP vs. low-FAP groups estimated by IOBR. (b) Heatmap showing fibroblast characteristics between high-FAP vs. low-FAP groups estimated by IOBR. (c) Pan-cancer analysis of FAP protein expression by IHC staining. Figure S2. (a) The transduction efficiency of 806-28Z CAR on splenic T cells derived from C57BL/6 was determined by flow cytometry. UTD cells served as negative controls. (b) The body weight of mice of each treatment group. All data are presented as the mean ± SEM of triplicate experiments. Figure S3. (a-d) In vivo experimental design. C57BL/6 mice were injected s.c. with PANC02-A2 cells and allowed to establish for 10 days. Mice were assigned to four experimental groups. Then, CAR-T cells were injected on day 10 (i.v.; n = 5 mice per group). Additional three mice in each group were used for harvesting tumor tissue during and after the CAR-T treatment. (b) The volume of tumors of each treatment group. (c) The body weight of mice of each treatment group. (d) CAR copy numbers in genomic DNA of residual tumors from each treatment group were measured by qRT-PCR (TaqMan probe). All data are presented as the mean ±SEM of triplicate experiments. *p < 0.05. Figure S4. Hematoxylin and eosin (HE) staining of important organs. Heart, liver, spleen, lung, and kidney were analyzed by HE staining. After study termination, the specimens were harvested from PANC02-bearing mice. The images were obtained under 200×magnification. The scale bar was 100 μm. The data shown are representative of experiments with similar results. Figure S5. Hematoxylin and eosin (HE) staining of important organs. Heart, liver, spleen, lung, and kidney were analyzed by HE staining. After study termination, the specimens were harvested from KPC1199-bearing mice. The images were obtained under 200×magnification. The scale bar was 100 μm. The data shown are representative of experiments with similar results. Fig [file 12967_2023_4080_MOESM1_ESM.doc]

**SUPPLEMENTARY Materials and methods**

**Mice and Animal experiment**

In the CAR-T single-dose treatment experiment, C57BL/6 mice were inoculated subcutaneously with CLDN18.2-positive PANC02 tumor cells (2×10^6^) on the right flank on day 0. Mice were then assigned to four experimental groups, including FAP-mBBZ, CLDN18.2-mBBZ, UTD, and the mixture of FAP-mBBZ and CLDN18.2-mBBZ. After the allograft tumors were established, 2×10^6^ for FAP-mBBZ, CLDN18.2-mBBZ, UTD T, and the mixture of FAP-mBBZ (1×10^6^) and CLDN18.2-mBBZ (1×10^6^) was infused to corresponding groups. Tumor dimensions and body weight were measured every 2–5 days. Tumor volume was measured by caliper and calculated by the formula V= (length*width2)/2.

**Quantitative Real-Time PCR**

mRNA was extracted from tumor tissue using Trizol reagent (Thermo Fisher Scientific). Subsequently, according to the manufacturer’s instructions, total RNA was reverse transcribed into cDNA (Promega). The quantitative real-time PCR reactions were performed using the following primers on ABI 7500 Real-Time PCR System (Applied Biosystems). 18s RNA was used as the internal control. Primers for CXCL12 were designed as F:5’GGTTCTTCGAGAGCCACATC and R:5’TCTTCAGCCG TGCAACAA. Primers for 18s RNA were designed as F:5’ CGGCTACCACATCCAAGGAA and R:5’ GCTGGAATTACCGCGGCT.

**Western Blot Analysis**

Cells were washed with PBS and then lysed with a protease inhibitor cocktail in RIPA buffer. The sodium dodecyl sulfate-polyacrylamide gel electrophoresis separated equal amounts of protein and transferred to polyvinylidene difluoride membranes (Millipore). 5% non-fat dry milk in PBS containing 0.1% Tween-20 blocked the membranes for 1h. Then, the membranes were exposed to the indicated primary antibodies in 5% non-fat dry milk in tris buffered saline Tween (TBST) at 4°C overnight. After washing and incubating with horseradish peroxidase (HRP)-conjugated secondary antibodies for 2h, Pierce ECL Western Blotting Substrate (Thermo Fisher Scientific) were incubated with membranes as the substrate of HRP, and membranes were scanned on film.

**SUPPLEMENTARY FIGURE**

**Fig.S1**


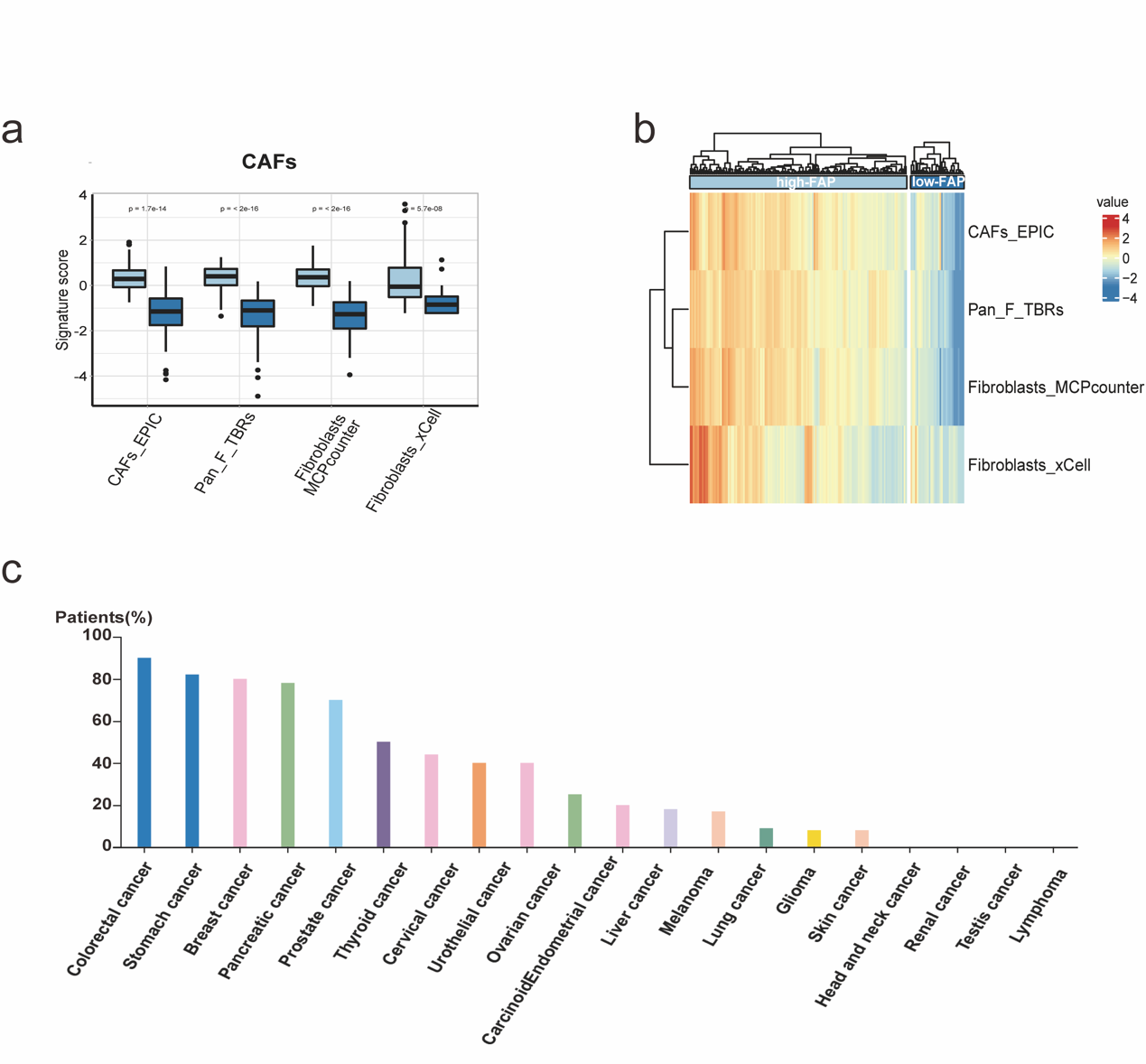


(a) Box plot comparing fibroblast characteristics between high-FAP vs. low-FAP groups estimated by IOBR. (b) Heatmap showing fibroblast characteristics between high-FAP vs. low-FAP groups estimated by IOBR. (c) Pan-cancer analysis of FAP protein expression by IHC staining.

**Fig.S2**


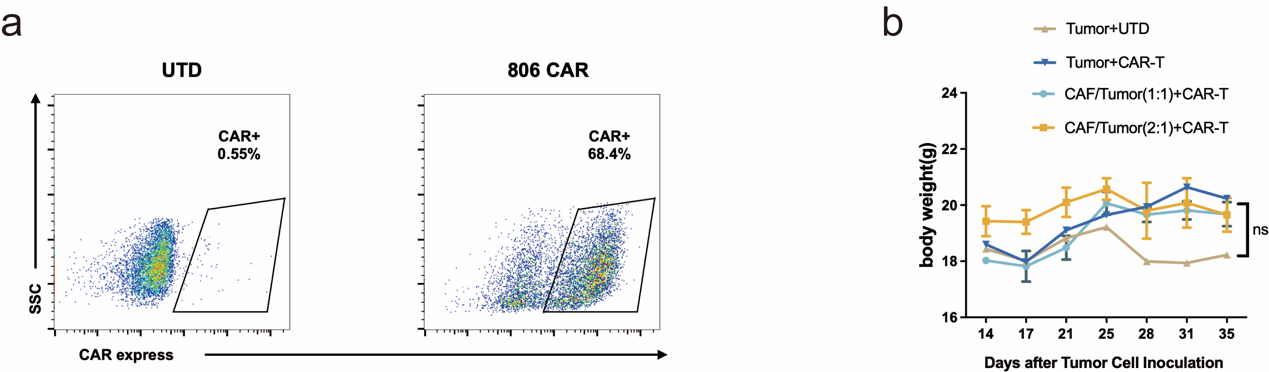


(a) The transduction efficiency of 806-28Z CAR on splenic T cells derived from C57BL/6 was determined by flow cytometry. UTD cells served as negative controls. (b) The body weight of mice of each treatment group. All data are presented as the mean ± SEM of triplicate experiments.

**Fig.S3**


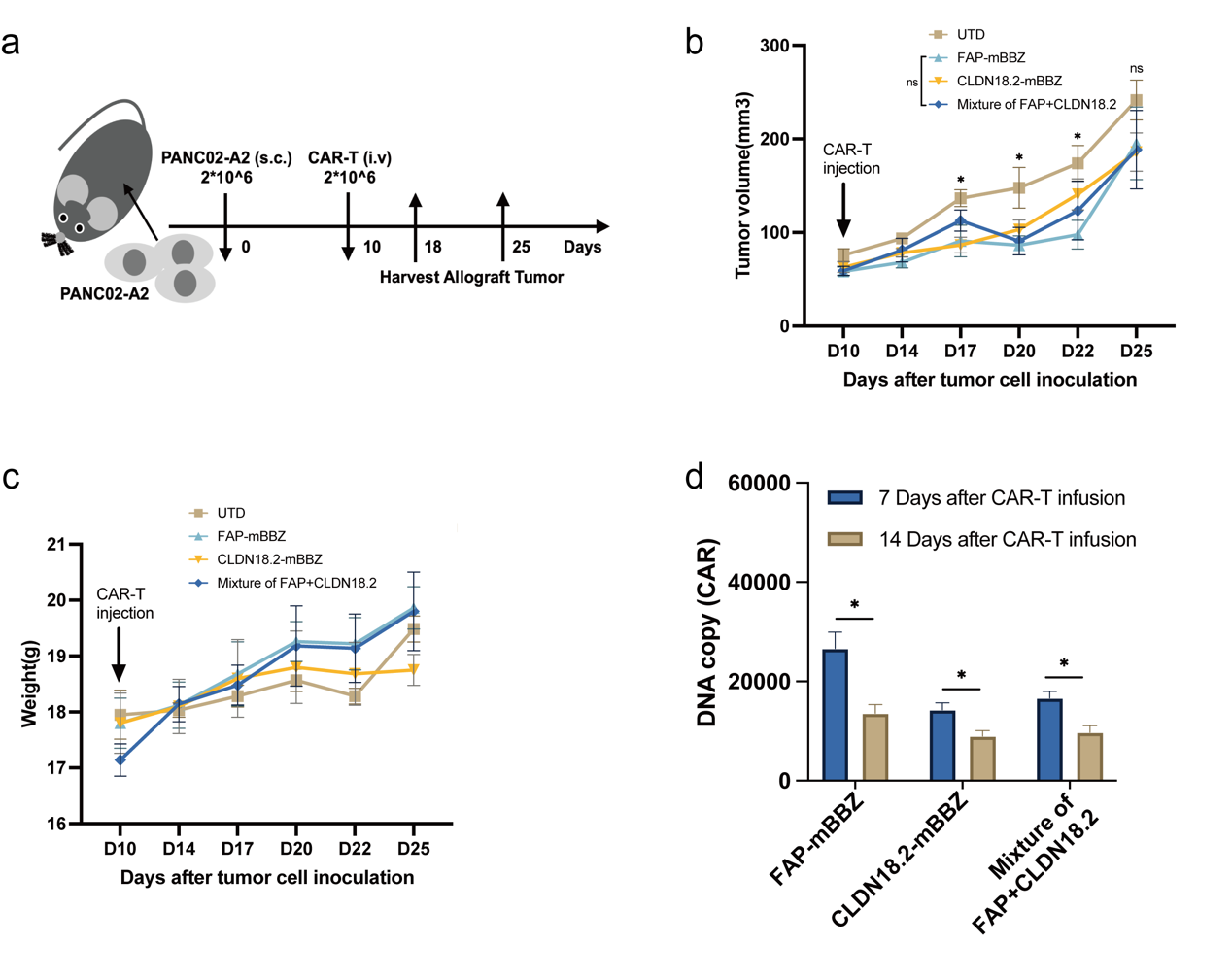


(a-d) *In vivo* experimental design. C57BL/6 mice were injected *s.c.* with PANC02-A2 cells and allowed to establish for 10 days. Mice were assigned to four experimental groups. Then, CAR-T were injected on day 10 (*i.v.*; n=5 mice per group). Additional three mice in each group were used for harvesting tumor tissue during and after the CAR-T treatment. (b) The tumor volume of tumors of each treatment group. (c) The body weight of mice of each treatment group. (d) CAR copy numbers in genomic DNA of residual tumors from each treatment group were measured by qRT-PCR (TaqMan probe). All data are presented as the mean ± SEM of triplicate experiments. **p* < 0.05.

**Fig.S4**


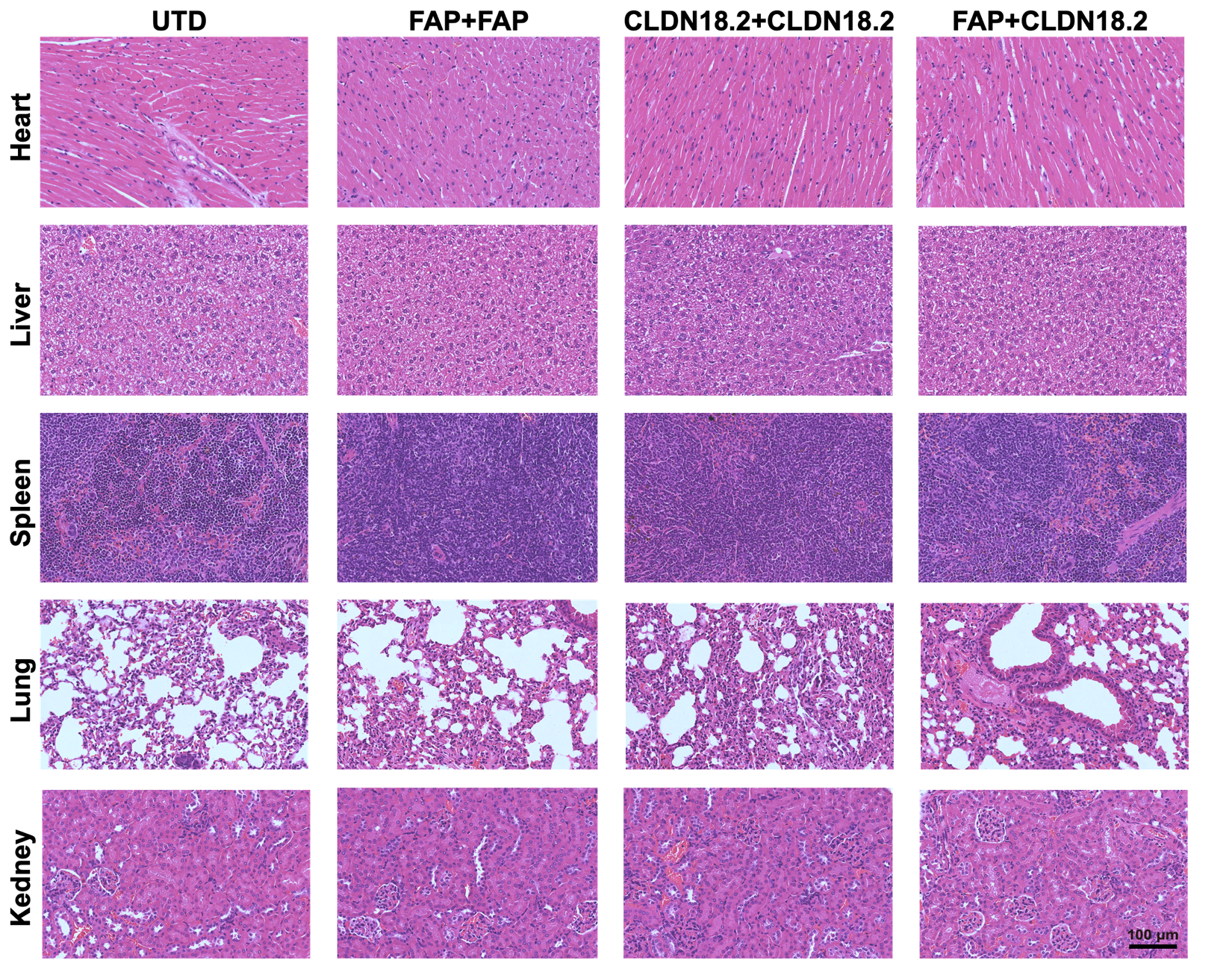


Hematoxylin and eosin (HE) staining of important organs. Heart, liver, spleen, lung, and kidney, were analyzed by HE staining. After study termination, the specimens were harvested from PANC02-bearing mice. The images were obtained under 200× magnification. The scale bar was 100μm. The data shown are representative of experiments with similar results.

**Fig.S5**

**
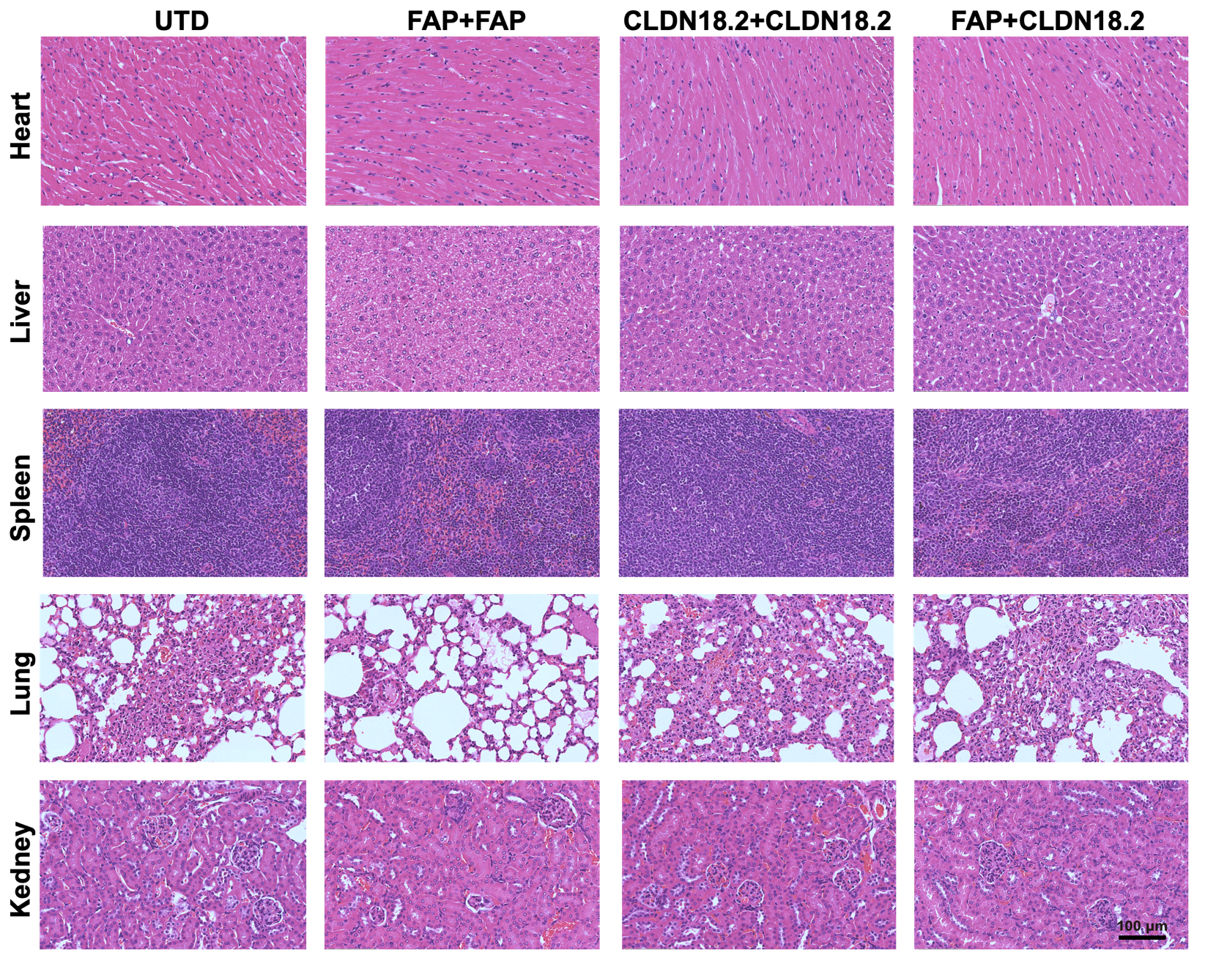
**

Hematoxylin and eosin (HE) staining of important organs. Heart, liver, spleen, lung, and kidney, were analyzed by HE staining. After study termination, the specimens were harvested from KPC1199-bearing mice. The images were obtained under 200× magnification. The scale bar was 100μm. The data shown are representative of experiments with similar results.

**Fig.S6**


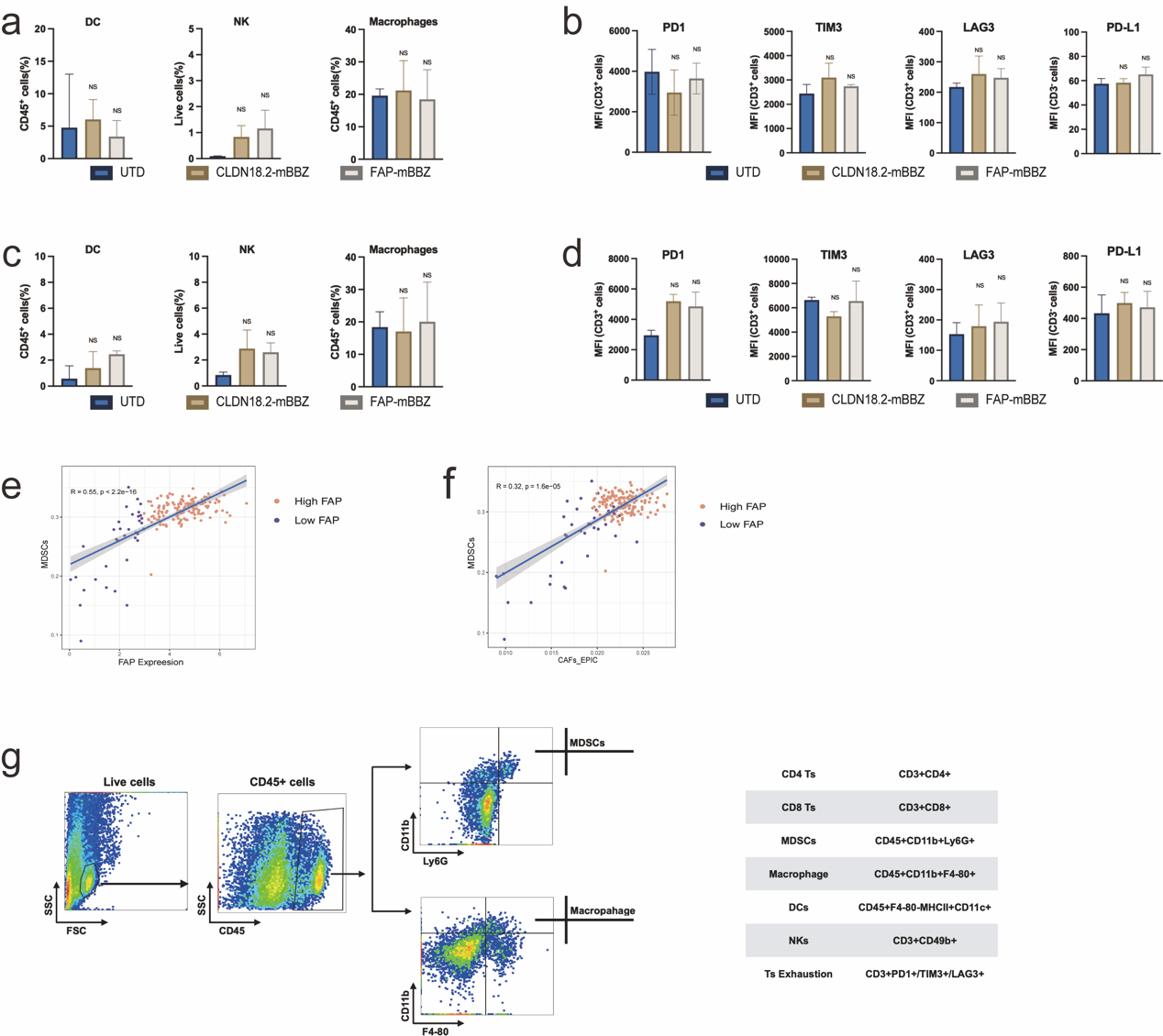


(a-b) Analysis of the immune cell in tumor tissues of PANC02-A2 xenografts. (a) Quantitation of tumor-infiltrating immune cells of each treatment group by flow cytometry. (b) Surface expression of Exhaustion marker in T cells and PD-L1 in tumor cells of each treatment group determined by flow cytometry. (c-d) Analysis of the immune cell in tumor tissues of KPC1199 xenografts. (c) Quantitation of tumor-infiltrating immune cells of each treatment group by flow cytometry. (d) Surface expression of Exhaustion marker in T cells and PD-L1 in tumor cells of each treatment group determined by flow cytometry. (e) Correlation between FAP expression and MDSCs characteristics estimated by IOBR in TCGA-PDAC patients. (f) Correlation between CAFs and MDSCs characteristics estimated by IOBR in TCGA-PDAC patients. (g) Gating strategies for flow cytometry. All data are presented as the mean ± SEM of triplicate experiments.
